# Clinical application of recommendations for neurobehavioral assessment in disorders of consciousness: an interdisciplinary approach

**DOI:** 10.3389/fnhum.2023.1129466

**Published:** 2023-07-12

**Authors:** Brooke Murtaugh, Amy Shapiro Rosenbaum

**Affiliations:** ^1^Department of Rehabilitation Programs, Madonna Rehabilitation Hospitals, Lincoln, NE, United States; ^2^Department of Brain Injury Rehabilitation, Park Terrace Care Center, Queens, NY, United States; ^3^TBI Model System, Icahn School of Medicine at Mount Sinai, New York, NY, United States; ^4^Brainmatters Neuropsychological Services, PLLC, Plainview, NY, United States

**Keywords:** brain injury, consciousness disorders (MeSH), diagnosis, prognosis, assessment practices

## Abstract

Accurate diagnosis, prognosis, and subsequent rehabilitation care planning for persons with Disorders of Consciousness (DoC) has historically posed a challenge for neurological care professionals. Evidence suggests rates of misdiagnosis may be as high as 40% when informal beside evaluations are used to determine level of consciousness. The presence of myriad medical, neurological, functional (motor, sensory, cognitive) and environmental confounds germane to these conditions complicates behavioral assessment. Achieving diagnostic certainty is elusive but critical to inform care planning, clinical decision making, and prognostication. Standardized neurobehavioral rating scales has been shown to improve accuracy in distinguishing between coma, unresponsive wakefulness syndrome/vegetative state and minimally consciousness state as compared to informal assessment methods. Thus, these scales are currently recommended for use as the informal “gold standard” for diagnostic assessment in DoC. The following paper will present an evidence-based approach to neurobehavioral assessment for use in clinical practice. Strategies for optimizing assessment and aiding in identification and management of confounds that can limit diagnostic accuracy will be provided. Finally, clinical application of an interdisciplinary approach to identifying and managing confounds will be discussed and how assessment results can be used to identify trends in performance and guide prognostic counseling with families.

## Introduction

Impairments in arousal and awareness after severe brain injury are ubiquitous to disorders of consciousness (DoC) which include coma, unresponsive wakefulness syndrome (UWS)/vegetative state (VS), and the minimally conscious state (MCS). MCS is a clinically heterogeneous category; as it is further stratified into MCS plus (+) and MCS minus (−). MCS+ is applied when observed behavioral responses demonstrate some level of preserved language functioning, as evidenced by the ability to follow commands, discriminate objects, or attempts to communicate (Bruno et al., 2011b; Giacino et al., 2022). Subtle behavioral differences that distinguish between these conditions are not easily detected on informal non-standardized bedside evaluation. For example, the re-emergence of spontaneous eye opening without evidence of purposive behavior is considered the hallmark of transition from coma to UWS/VS, yet even patients in MCS+ may demonstrate poor sustained arousal and highly inconsistent purposive behaviors. Conversely, reflexive vocalizations, eye, and limb movements are all commonly seen in UWS/VS, and may be misinterpreted as purposive responses to stimuli. Expected variability and ambiguity of behavioral responses further complicates the clinical phenotype and limits diagnostic certainty at the individual level. Consequently, informal bedside assessment and team consensus carries a 40% misdiagnosis rate (Schnakers et al., 2009; Wang et al., 2020). The potential consequences of misdiagnosis are great, as one’s level of consciousness plays a central role in driving important care decisions such as withdrawal of life sustaining treatments and access to specialty post-acute and rehabilitation services.

This paper will present a structured approach to evidence-based assessment of DoC to apply clinically to improve diagnostic accuracy across the continuum of care. An overview of practice guidelines and program recommendations will be provided, which include the use of standardized neurobehavioral rating scales to reduce diagnostic error. Common confounds germane to DoC will be discussed along with strategies to help address and mitigate their impact on behavior responsiveness and optimize diagnostic certainty. The clinical strategies to neurobehavioral assessment highlighted in this paper were included based on published evidence including the American Academy of Neurology (AAN) DoC Practice Guideline Recommendations (Giacino et al., 2018) and European Academy of Neurology Guidelines for Diagnosis of Coma and DoC (Kondziella et al., 2020) in conjunction with various evidence-informed recommendations such as the American Congress of Rehabilitation Medicine’s DoC Minimal Competency Recommendations (Giacino et al., 2020a). Specifically, published Guidelines and Recommendations underwent intensive expert investigation, systematic review, data analysis, application of the Grading Recommendation Assessment, Development and Evaluation (GRADE) process. Additionally, the AAN DoC Guideline development applied the AAN Clinical Practice Guideline Process Manual to direct the methods of creating the 2018 Practice Recommendations.

## Neurobehavioral assessment: DoC practice guidelines and recommendations

Published American and European DoC practice guidelines and American minimal competency recommendations for rehabilitation programs support the use of valid and reliable standardized neurobehavioral rating scales as the “gold standard” for assessment of persons with DoC (Giacino et al., 2018, 2020a; Kondziella et al., 2020). Their superior diagnostic accuracy as compared to team-based consensus has been supported through the past published evidence (Schnakers et al., 2009; Wang et al., 2020). Patient performance on these scales can assist in identifying level of consciousness within the DoC spectrum, facilitate detection of diagnostic confounds and guide development of strategies aimed at accessing latent cognition to maximize rehabilitation potential and functional outcomes. Moreover, serial assessments can be used to identify trends in the rate and trajectory of recovery that can help inform prognosis and degree of long term disability (Giacino et al., 2018, 2020a). Tables 1, 2 present a complete list of practice guidelines and program recommendations related to diagnostic assessment.

**TABLE 1:** American (AAN) and European (EAU) DoC practice guideline recommendations addressing neurobehavioral assessment.

Recommendations to improve diagnostic accuracy	● Use standardized serial assessment deemed reliable by American Congress of Rehabilitation Medicine (See Seel et al., 2010; AAN; Also see Table 2).
	● Reassessment intervals dependent upon patient presentation (AAN)
	● Optimize patient arousal prior to assessment, especially when observed to be diminished (AAN)
	● Use of mirror to diagnose visual pursuit (EAN).
	● Observe for spontaneous motor behaviors to diagnose signs of consciousness (EAN).
	● Use of FOUR consciousness assessment in ICU (EAN).
	● Use of CRS-R for consciousness assessment in subacute and ICU (EAU).
Recommendations to mitigate diagnostic confounds	● Use multi-modal assessment tools when bedside assessment results are unclear (AAN).:
	● Utilize serial assessment results to identify and address complications (AAN).
	● Use of PET, FMRI, EEG to identify covert consciousness and differentiate between UWS/MCS (EAN).
Recommendations related to prognosis	● Utilize serial standardized assessment inform prognosis (AAN).
	● Use CRS-R to inform prognosis with non-traumatic vegetative state presentation (AAN).
	● EAU does not provide Guidelines addressing prognosis.

Adapted from Giacino et al. (2018), Kondziella et al. (2020).

**TABLE 2:** Minimal competency recommendations for programs serving DoC population: recommendations related to neurobehavioral assessment.

Recommendations to improve diagnostic and prognostic accuracy	● Specialized programs should adopt a systematic approach to diagnostic and prognostic assessment. ● Protocols should be in place to reduce misdiagnosis and mitigate confounds Validated measures should be used to monitor recovery trajectory from baseline assessment
Recommendations to mitigate diagnostic confounds	● Upon admission, a comprehensive neurosensory exam should be conducted to identify any unidentified auditory, visual, motor or somatosensory deficits Address environmental factors that may influence arousal and patient performance

Adapted from Giacino et al. (2020b).

## Overview of standardized neurobehavioral assessments for DoC

There are several evidence-supported standardized behavior scales that can be employed in clinical practice, at all levels of care, to aid diagnosis, prognosis and family counseling for DoC. Irrespective of specific scale used, assessment of persons with DoC typically evaluates behavioral responsiveness in the common domains of sensory process and function including: auditory, visual, motor, oral motor, communication and arousal (Kalmar and Giacino, 2005; Pape et al., 2009, 2014; Morrissey et al., 2018). Often responses are graded based on a hierarchy of behaviors that demonstrate neurological functioning at either a brainstem, subcortical or cortical level (Giacino et al., 2022). Seel et al. (2010) conducted a review of available behavioral DoC assessment scales and provided recommendations for use based on the psychometric qualities (validity and reliability) of each scale and other criteria. The Coma Recovery Scale-Revised (CRS-R) was the only tool recommended for clinical use with minor reservations secondary to its strong reliability, validity, standardized administration and scoring procedures, interpretative scoring guidelines, and ease of accessibility for clinicians. Five additional scales were recommended for practice with moderate reservations including the SMART, WNSSP, SSAM, WHIM, and DOCS. One scale, the CNC, was recommended, however, with major reservations. Four scales were specifically not recommended for bedside assessment of DOC due to poor validity, reliability or a lack of standardization. These included the RLS85, LOEW, and CLOCS (Seel et al., 2010). See Table 3.

**TABLE 3:** Recommended behavioral assessment scales: Pros & Cons comparison of utilization.

Assessment Scale	Pros	Cons	Recommendation of use
CRS-R	● Freely available	● Unstudied prognostic validity	Minor reservations
	● Valid and reliable for VS/MCS/EMCS		
	● Standardized administration and scoring		
	● Reasonable time to administer		
SMART	● Defined administration and scoring	● Requires purchase	Moderate reservations
	● Content validity for VS/MCS/EMCS	● Completion of 5 day training course	
	● 60 min to complete		
WNSSP	● Excellent internal consistency	● Approx. 45 min to administer	Moderate reservations
	● Content validity for VS/MCS/EMCS	● Unproven prognostic validity	
SSAM	● Defined administration and scoring	● Absent diagnostic validity studies	Moderate reservations
	● Reasonable time to administer	● Lacks evidence for test-retest reliability and internal consistency	
	● Content validity for VS/MCS/EMCS		
WHIM	● Defined administration and scoring	● Requires purchase	Moderate reservations
	● Content validity for VS/MCS/EMCS	● Approx. 60 min to administer	
DOCs	● Defined administration and scoring	● Unproven inter-rater reliability and test-retest reliability	Moderate reservations
	● Reasonable time to administer		
	● Acceptable content validity.		

Adapted from Seel et al. (2010).

Since the review Seel et al. (2010), the CRS-R has undergone further extensive investigation. Bodien et al. (2016) performed sensitivity and specificity analyses using CRS-R derived diagnoses to determine that a total cut-off score of eight or higher reliably distinguishes between patients in UWS/VS and MCS in 93% of cases (Bodien et al., 2016). Collective evidence evaluating the utility of the CRS-R, compared to other behavior rating scales, diagnostic modalities, and neurophysiological studies, demonstrates the superiority of the CRS-R as a sensitive and reliable tool to accurately identify and discriminate among the levels of DoC (Lechinger et al., 2013; Annen et al., 2019; Formisano et al., 2019a; da Conceição Teixeira et al., 2021). Additional evidence focusing on the utility of the CRS-R identifies the benefit of serial use of the CRS-R to improve accuracy of identifying behavioral presentation of DoC (Wannez et al., 2017; Yang et al., 2021). Further evaluation and investigation of the CRS-R has produced development of a CRS-R index to improve total score interpretation and translation of the CRS-R into multiple languages for international use (Lombardi et al., 2007; Tamashiro et al., 2014; Binder et al., 2018; Annen et al., 2019; Zhang et al., 2019). Moreover, research also supports the use of the CRS-R to help inform the trajectory of DoC recovery and prognosis at the individual level (Bodien et al., 2016, 2022; Giacino et al., 2018, 2020a).

## Assessment of consciousness in the intensive care unit

Standardized behavior rating scales such as the CRS-R are rarely utilized in the intensive care unit (ICU) for diagnostic assessment of Doc after severe brain injury (Chaturvedi et al., 2021). Time demands imposed by these tools, along with use of sedation, paralytics, mechanical ventilation and movement restricting equipment all serve as practical barriers to the implementation of standardized assessment of consciousness in DoC patients (Chaturvedi et al., 2021). Consequently, physicians in the neurological ICU routinely perform non-standardized bedside evaluations to determine level of consciousness.

The Glasgow Coma Scale (GCS) is the most widely known and utilized tool for assessing brain injury severity and level of coma in ICU/acute care settings due to its feasibility and time efficient implementation required at this level of care (Formisano et al., 2019b; Helbok et al., 2022). However, the GCS is an observational scale and lacks sensitivity to distinguish among different levels of consciousness, and to identify salient features of MCS (−/+) in particular (Bodien et al., 2021). Bodien et al. (2021) compared GCS score combinations to CRS-R scores and found great variability and diagnostic error rates when the GCS is used to identify consciousness. Specifically, they found that GCS total scores did not differentiate among DoC subtypes and that when GCS scoring criteria are used, many persons in MCS were erroneously classified as being “comatose.” The Full Outline of UnResponsiveness (FOUR) is an additional neurological assessment implemented in the ICU that is recommended by the European (EU) DoC guidelines for assessment of level of consciousness in the ICU (Kondziella et al., 2020). The EU recommends the use of the FOUR over the GCS in light of its convenience of serial use by clinicians and nurses. Additionally, the FOUR is more sensitive in capturing certain MCS and locked-in syndrome behaviors involving eye movement which decreases the risk of misdiagnosis (Bruno et al., 2011a; Kondziella et al., 2020; Bodien et al., 2021). Although the FOUR is a recommended assessment for this patient population, there are currently efforts underway to develop and validate an abbreviated version of the CRS-R and other standardized rating scales adapted for DoC patients in the ICU (Aubinet et al., 2021; Bodien et al., 2021; Sanz et al., 2021).

## Neurobehavioral assessment across care settings: impact of confounds on diagnostic accuracy

Notably, even standardized behavioral rating scales are limited in their ability to differentiate a subset of ICU patients at risk for being misidentified as having a DoC due to the presence of related clinical features such as complete motor paralysis or language impairment (Kondziella et al., 2020). Recent research has found approximately 15–20% of persons classified as having a DoC in the ICU actually have cognitive motor dissociation (CMD), a condition of covert consciousness characterized by the retained capacity for volitional thought in the absence of overt behavioral manifestations or motoric output (Schiff et al., 2005; Owen et al., 2007; Owen and Coleman, 2008). CMD can only be detected with the use of advanced technologies such as functional MRI and electroencephalograph (EEG). These modalities have demonstrated the ability to identify cases of higher-order cortex motor dissociation by eliciting accurate responses to language and music based tasks in persons behaviorally presenting as UWS/VS (Edlow et al., 2017; Claassen et al., 2019; Kondziella et al., 2020; Thibaut et al., 2020). Active and passive paradigms in using fMRI and EEG have demonstrated utility in identifying CMD in behaviorally unresponsive patients. However, it has been found that passive paradigms have a greater likelihood of capturing preserved consciousness (Kondziella et al., 2016; Aubinet et al., 2022). The scientific understanding of CMD is evolving, but current evidence suggests it is likely a distinct phenomenon separate from the DoC spectrum (Kondziella and Stevens, 2022), more akin to a functionally locked-in syndrome. Evidence suggests those who are identified as CMD while in the ICU have an improved functional recovery as compared to those unresponsive patients who demonstrate no evidence of consciousness with advanced neuroimaging (Edlow et al., 2017, 2021). This is a critical issue, given detection of consciousness, or lack thereof, can have significant impact on surrogate decisions regarding withdrawal of care while in ICU (Giacino et al., 2018; Graham et al., 2018; Naci and Owen, 2022; Pruvost-Robieux et al., 2022).

Beyond CMD, persons with DoC present with a wide range of overlying complications and comorbidities that can exacerbate the complexity of the clinical picture (Majerus et al., 2009; Schnakers et al., 2015; Bodien et al., 2022). US practice guidelines recommend that prior to making a final determination regarding level of consciousness, efforts be made to identify and treat confounding conditions that impede accurate diagnosis and directly impact the ability to actively participate and interact with others (Giacino et al., 2018). Similarly, minimal competency recommendations (Giacino et al., 2020b) state that rehabilitation programs should have a protocol in place to detect and treat confounds that can mask evidence of conscious awareness and lead to misdiagnosis. For purposes of the present paper, the authors conceptualize these confounds within three primary categories: medical/neurological issues, overlying functional (motor/sensory/cognitive) impairments, and adverse environmental influences on behavior responsiveness (see Table 3). Some confounds may be present in the acute/ICU setting, whereas others may not develop or become apparent until the post-acute setting.

### Medical confounds

Patients with DoC are at risk of developing medical complications with a frequency that contributes to high rates of re-hospitalization (Whyte and Nakase-Richardson, 2013). Common medical and neurological confounds include secondary complications such as hydrocephalus, seizures, secondary hemorrhage or intracranial fluid collection, cerebral edema, increased intracranial pressure, infections (pneumonia, urinary tract infections, sepsis), sleep disorders, metabolic/endocrine disturbances and other systemic comorbidities (Ganesh et al., 2013; Whyte and Nakase-Richardson, 2013). The occurrence of one or more medical complications may suppress a person’s level of responsiveness during standardized assessment. Additionally, an increased number and frequency of comorbid conditions and complications has been associated with a protracted trajectory of recovery and worse long-term outcomes (Whyte and Nakase-Richardson, 2013).

### Functional confounds

Functional confounds include impairments that negatively affect the patient’s ability to demonstrate motor output, integrate sensory information, or otherwise provide appropriate responses to test stimuli. Beyond conditions like CMD, common motor confounds to consider include spasticity and joint contracture. Spasticity is a frequent confound that many patients with DoC experience; reported incidence rates are as high as 90% (Martens et al., 2017; Zhang et al., 2021b). Other motor confounds include hemiplegia/hemiparesis, concomitant spinal cord injury, myopathies, neuropathies, dystonia and other central nervous system movement disorders. Sensory and perceptual confounds such as vision, hearing, or other impairments may occur due to damage to the peripheral sensory nerves, cranial nerves, thalamus, primary sensory cortices, or cortico-sensory pathways. Cognitive confounds include overlying aphasia, apraxia, agnosia, problems with higher level auditory or visual processing, as well as disorders of diminished drive and motivation (Lancioni et al., 2010, 2012).

### Environmental confounds

Environmental confounds are controllable factors that should be systematically evaluated for their impact on patient arousal and overall level of responsiveness. Sleep-wake cycle and concomitant arousal disturbances are intrinsic to DoC, but can be exacerbated by inappropriate lighting, ambient noise, or the sedating effects of commonly used medications for seizures, pain and spasticity. Other potential variables include conditions such as time of day, patient positioning, and the presence of physical restraints (e.g., splints, casts, braces) that may impede the ability to demonstrate purposive motor responses. In addition, pain and discomfort, extreme room temperature, excessive stimulation, and the presence of distracting or competing stimuli may limit attention capacity and ultimately impact validity and reliability of assessment (Giacino et al., 2020a; Bodien et al., 2022) (see Table 4).

**TABLE 4:** Common possible confounds seen in DoC population.

Aphonia
Concomitant spinal cord injury
Contractures
Excessive stimulation
Fractures
Hemiplegia/Paresis
Hydrocephalus
Intracranial complications
Illness/Infection
Lighting
Medication side effects
Myopathies
Movement disorders
Neuro-endocrine dysfunction
Neuropathies
Noise
Paroxysmal autonomic hyperactivity
Patient positioning
Presence of restraints
Seizures
Sleep disorders
Spasticity
Temperature
Time of day

## Practical strategies for optimizing neurobehavioral assessment across care settings

### Interdisciplinary assessment

Effective neurobehavioral assessment begins with an interdisciplinary approach that promotes coordination, collaboration and communication among professionals across care specialties including medical, nursing and rehabilitation. Baseline measures of performance on behavior rating scales should be obtained by multiple disciplines, in different environments at different times of day, and under different conditions to establish trends in arousal and response patterns and aid in comparing and analyzing any scoring inconsistencies. Assessment schedules can become more individualized over time once conditions of optimal arousal and responsiveness are identified. Standardized neurobehavioral assessment can be administered by a variety of care specialists including physicians, neuropsychologists, speech, occupational and physical therapists across the care continuum. A general rule, assessments should be performed by clinicians who have experience working with persons with DoC and received specialized training in the tool being utilized. Findings from a physician survey suggest lack of knowledge and skill are practical difficulties contributing to poor implementation of the CRS-R (Chaturvedi et al., 2021). In contrast, a study by Løvstad et al. (2010) found that increased experience administering the CRS-R increased the reliability of assessment results, emphasizing the importance of providing systematic interdisciplinary education and training in DoC assessment. A staff training curriculum should include an overview of DoC, introduction to neurobehavioral assessment of DoC, and hands-on training to ensure a consistent standard of care and implementation across disciplines (Giacino et al., 2020a). Clinical training and mentorship should also provide clinicians ample opportunities to practice test administration and scoring on a wide range of DoC patients with varying behavioral presentations.

### Medical confounds

Promoting medical stability is key to optimizing neurobehavioral assessment. Systematic medical monitoring helps ensure early detection and treatment of comorbidities or complications that may arise (Zhang et al., 2021a). Brain imaging studies, including CT and MRI, should be performed and reviewed on admission to a post-acute setting to screen for potential neurological confounds or complications (Giacino et al., 2020a). Efforts should be made to reduce or eliminate the use of potentially sedating medications where possible at any level of care when standardized neurobehavioral assessment is implemented. Additionally, nursing initiating systematic sleep monitoring can facilitate timely management of sleep wake issues including introducing the strategic use of medications to promote improved nighttime sleep and daytime arousal to optimize assessment (Giacino et al., 2020a; Gottshall and Rossi Sebastiano, 2020). A comprehensive neurosensory examination can identify the presence of previously unrecognized overlying motor, sensory, or cognitive impairments. This may involve testing of reflexes, cranial nerve assessment, and/or the use of sensory evoked potentials to evaluate the integrity of primary sensory systems, peripheral nerves, and to obtain information about cortical signaling and processing (De Salvo et al., 2015). Pain perception may be difficult to identify in persons with DoC, yet pain should be treated for patient comfort (Fins and Bernat, 2018; Giacino et al., 2018). The EU guidelines include a recommendation of the use of the Nociception Coma Scale-Revised (NCS-R) to monitor for signs of pain and discomfort in persons with DoC (Kondziella et al., 2020). The NCS-R is a behavior assessment tool that was developed to assess pain perception in patients with DoC (Schnakers et al., 2010; Chatelle et al., 2012, 2016b) and can be used to aid prompt utilization of pain management strategies.

### Functional confounds

Functional confounds may first be suspected during initial assessment by neuropsychology, occupational or speech therapy. Development of adaptation strategies to functional confounds requires collaboration and application across disciplines in order to be effective. For example, a combination of nursing, rehabilitation, pharmacologic and surgical interventions may be required to maintain joint integrity and assuage spasticity, pain or contractures to support enough range of motion to elicit active movement. Some motor impairments may benefit from adapting test administration procedures to facilitate the ability to respond. Suspected hemiparesis on the affected side would warrant presentation of stimulus on the unaffected side. Providing proximal support at the elbow to help a person compensate for limb weakness and perform functional object use. Similarly, presenting visual stimuli in a vertical format to one side of a patient’s visual field to help accommodate for a gaze deviation, suspected hemispatial neglect, or visual field loss as an adaptation for vision changes. Another common adaptation is determining the best compensatory response mode (e.g., head/mouth control switch or eye gaze) for a person with severe motor limitations, and subsequently implementing an augmentative technology to aid communication and environmental control. Finally, offering increased time to respond may facilitate detection of command following in persons with slow speed of auditory processing, sensory or perceptual impairments, or suspected drive state disorders.

As a supplementary tool, the updated CRS-R manual (Giacino et al., 2020b) includes a test completion coding system to help clinicians identify and characterize factors that may have impacted response validity during any given assessment. These codes allow for the documentation of suspected or known patient specific confounds of the patient as well as extraneous factors that may have affected a patient’s score in a specific sub-scale or the total CRS-R score. In addition, Chatelle et al. (2016a) identified nine impossible and 36 improbable CRS-R sub-score combinations that can be used to aid response interpretation and ensure accuracy of obtained CRS-R scores. Each improbable sub-score combination is accompanied by a list of possible contributing factors to consider when scoring errors are ruled out (Chatelle et al., 2016a).

### Environmental confounds

Environmental adaptation, based on individual need can enhance the ability to participate with interpersonal interactions. Attempts to increase arousal should be undertaken prior to initiating an assessment and anytime arousal is observed or suspected to be diminished throughout the evaluation (Giacino et al., 2018, 2020a). The CRS-R administration manual includes a structured arousal facilitation protocol that provides a good model for eliciting and promoting sustained arousal during assessment (Kalmar and Giacino, 2005). Prior to initiating the assessment, ensure proper head and limb positioning, remove splints or braces if feasible, and observe the patient for any signs of pain or discomfort. Accommodate for any other potential limiting environmental conditions such as timing of assessment as it relates to medication dosing, lighting, temperature, and noise levels.

### Serial assessment

Serial monitoring over time, using recommended neurobehavioral assessment tools, can facilitate early detection of behaviors that may indicate emerging awareness and guide development of individualized rehabilitation strategies. Collated results from repeat assessments performed over time, can assist in ensuring accuracy of initial diagnosis, monitoring trends in recovery and maximizing detection of the patient’s highest level of function over time (Bagnato et al., 2017; Lee et al., 2020; Nekrasova et al., 2021; Bodien et al., 2022). One-time use of standardized neurobehavioral assessment can fail to capture purposive behaviors demonstrated infrequently. Current practice guidelines and recommendations do not specify how often serial examination should be performed. Rather, they state that the frequency of reassessment should be determined based on individual circumstances (e.g., extent of variability in arousal and responsiveness, the presence of confounds), but be sufficient to address individual specific questions of interest (Giacino et al., 2018, 2020a). Emerging research exemplifies how the number of repeated administrations of the CRS-R can significantly influence the clinical diagnosis. Wannez et al. (2017) performed the CRS-R on a sample of 123 patients with chronic DoC at least six times within a 10-day period. They found that diagnoses made based on a single CRS-R led to a misdiagnosis rate of 36% as compared to diagnoses constructed on multiple CRS-R trials. Based on these results, the authors recommend performing at least five assessments within a short time interval (e.g., 2 weeks) to boost diagnostic certainty, even in persons with chronic DoC (Wannez et al., 2017). A similar study by Yang et al. (2021) developed statistical formulas to estimate the probability of positive response with use of the CRS-R in relation to the minimal number of successive examinations. Yang et al. (2021) identified that a minimum of five assessments is needed for patients with non-traumatic DoC and six assessments for traumatic DoC (Yang et al., 2021).

### Multimodal assessment

A multimodal approach to assessment should be employed to improve sensitivity and specificity of assessment results, thereby improving diagnostic accuracy (Majerus et al., 2005; Giacino et al., 2006; Owen et al., 2007; Coleman et al., 2009). If available and feasible, the use of advanced technologies can help enhance diagnostic certainty, especially in cases where behavioral responses remain ambiguous or infrequent despite serial behavior assessment, or when confounds to valid assessment are identified (Giacino et al., 2018, 2020a). Functional MRI, positron emission tomography, single photon-emission computed tomography, electroencephalography and evoked potentials have all demonstrated utility in detecting cover evidence of awareness not demonstrated on serial bedside behavior exam such as in cases of CMD mentioned earlier (Edlow et al., 2017, 2021; Claassen et al., 2019; Kondziella et al., 2020; Thibaut et al., 2020). While advances in these technologies hold promise for improving diagnostic certainty, especially in cases of CMD, unfortunately these tools are not readily available for routine clinical use as it stands today. Additional elements of a multimodal approach to neurobehavioral assessment include: results of objective tests, performance on standardized behavior scales, family and staff reports. Individualized Quantitative Behavioral Assessment (IQBA) is an adjunctive assessment strategy that may be helpful in cases where observed behavior and performance on standardized rating scales are ambiguous. IQBA can be used to address specific questions in a standardized manner to assist in identifying and improving confidence in determining level of consciousness (Whyte et al., 1999; Day et al., 2018; Giacino et al., 2020a).

**FIGURE 1:**
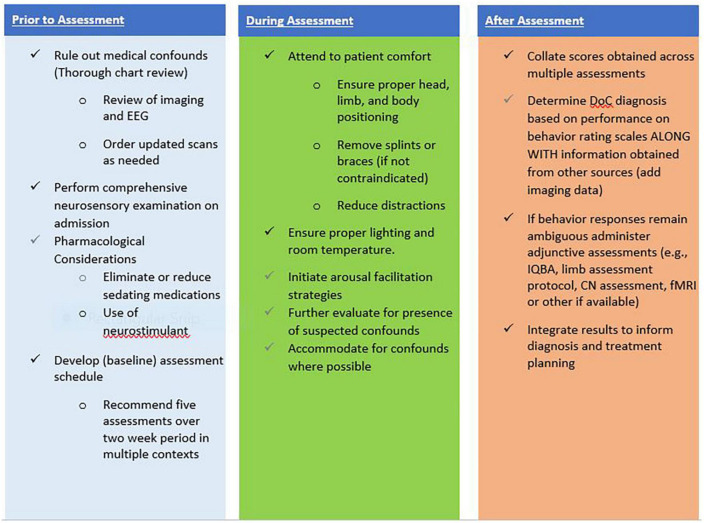
Checklist suggested for optimizing neurobehavioral assessment.

As patients progress through the DoC continuum toward emergence, a range of validated measures should be used to monitor progress across multiple domains (e.g., arousal, mobility, communication, participation). As performance reaches a ceiling on standardized behavior rating scales such as the CRS-R, measures capable of capturing more complex abilities should be employed (Giacino et al., 2020a). Although outside the scope of this paper, there are tools available for assessing agitation, confusion, attention, orientation, language and communication in persons with DoC demonstrating MCS+ or emergence behaviors. These assessments can include the Confusion Assessment Protocol, Agitated Behavior Scale, Orientation Log and the Loewenstein Communication Scale (Bogner et al., 1999; Sherer et al., 2005; Frey et al., 2007; Spiteri et al., 2021; Aubinet et al., 2022). Figure 1 presents an overview of recommended strategies to optimize the patient and environment to ensure accuracy of assessment results.

## Neurobehavioral assessment informing prognosis and guiding family counseling

**FIGURE 2:**
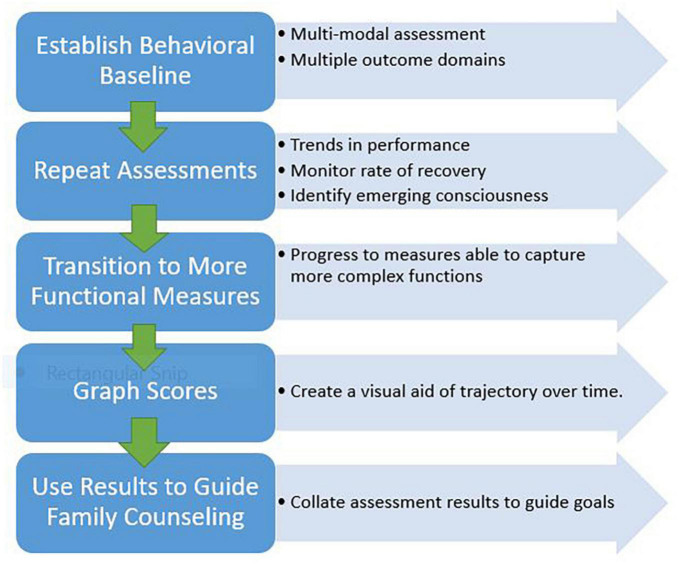
Recommended sequential approach to serial assessment application and outcome monitoring.

Serial monitoring over time, using recommended neurobehavioral assessment tools, can facilitate early detection of behaviors that may indicate emerging awareness and thus guide development of individualized rehabilitation strategies. Collated results from repeat assessments can help identify trends in recovery that can inform long-term prognosis for persons with DoC. A compendium of evidence supports the prognostic utility of CRS-R scores and the trajectory of those scores over time to predict recovery of consciousness and functional outcome (Pignat et al., 2016; Portaccio et al., 2018; Annen et al., 2019; Lucca et al., 2019; Hamilton et al., 2020; Boltzmann et al., 2021). Ultimately, when results are to be used to help inform prognosis, serial CRS-R scores must be considered along with other significant factors such as patient age, premorbid conditions, injury comorbidities and severity, frequency of complications and effective acute management (Estraneo et al., 2018; Steppacher et al., 2020; Kowalski et al., 2021; Nekrasova et al., 2021; Siegert et al., 2022). Figure 2 presents a recommended structured approach to applying serial assessment to outcome monitoring.

Ongoing tracking of scores over time provides objective data that can be used to help guide family education and counseling efforts regarding clinical care decisions and long term care planning. When communicating diagnosis and prognosis with family caregivers, rely on use of simple language that is easy to understand, and provide periodic updates (Giacino et al., 2020a). Counseling should include education about their loved one’s behavioral assessment results, information about the assessment tools used and how obtained results relate to expectations for recovery. Presenting a graph of scores on the CRS-R and other measures throughout the course of care is a useful tool to visually demonstrate a person’s recovery trajectory and areas of progress (or lack thereof). This approach to counseling is aimed at helping family caregivers understand their loved one’s condition and care needs so they can develop realistic expectations for recovery and collaboratively establish an appropriate short- and long-term plan of care (Giacino et al., 2020a).

## Future of DoC assessment

Assessment of Doc is rapidly evolving. As previously mentioned, there are efforts underway to develop and validate consciousness screens and short-form versions of existing scales to facilitate expedient, accurate assessment in critical care settings. Additionally, a valid and reliable DoC assessment in young children is needed. Slomine et al. (2019) have developed the Coma Recovery Scale for Pediatrics (CRS-P) to evaluate DoC in children 12 months and older. The CRS-P is undergoing continued investigation related to strength of psychometric properties and utility of use in the pediatric DoC population (Slomine et al., 2019). Ongoing exploration into ways to expand the use of neuroimaging and electrophysiological technologies to aid detection of consciousness and to identify CMD early post injury is a high priority to better inform medical decision-making (e.g., withdrawal of care) and overall care planning. Brain Computer Interface (BCI) is an additional modality that has been studies extensively as an assessment tool to identify consciousness or CMD through “cerebral communication” (Farisco et al., 2014; Ortner et al., 2017; Wang et al., 2017). BCI is evolving through research and ideally will become a clinical tool feasible for utilization at the bedside. Evaluating the comparative sensitivity, specificity, cost, and overall ease of implementation among these technologies will help direct future efforts to make these tools more accessible. There is a significant need to develop a prognostic algorithm where neurobehavioral assessment results, in combination with evidence-based biomarkers (e.g., neuroimaging, electrophysiological studies, etc.) can be applied to promote diagnostic accuracy and enhance the precision of prognostic estimates to support informed care decisions (Hammond et al., 2021; Mainali et al., 2022; Olson et al., 2022). Finally, operationalizing an interdisciplinary education, training, and mentorship methods can help ensure reliability and validity of assessment results and enhance clinical application of results to guide quality DoC care.

## Conclusion

Standardized neurobehavioral assessment is a primary feature of quality DoC care essential to ensuring diagnostic accuracy, appropriate rehabilitation planning, and outcome monitoring. Given the high prevalence of medical, neurological, functional and environmental confounds in persons with DoC, it is imperative to have tools that facilitate accurate bedside assessment of consciousness. Evidence-based neurobehavioral rating scales are widely available and accessible tools for bedside use across the continuum of care. Serial and multimodal assessment can improve diagnostic certainty, identify trends in recovery over time, and guide prognostic counseling with families. As technology continues to advance through future funding and research, the application of multimodal assessment tools will likely continue to evolve and play an increasingly important role in supporting DoC assessment and overall care planning for this population.

## Author contributions

BM and AS equally contributed to the development and completion of manuscript content, including review of the literature, manuscript prose, development of tables and figures, and the editing process. They completed the final review of the manuscript together over various virtual meetings and achieved consensus on final manuscript and submission. Both authors contributed to the article and approved the submitted version.
